# *Clostridium difficile* toxin B induces autophagic cell death in colonocytes

**DOI:** 10.1111/jcmm.13555

**Published:** 2018-01-31

**Authors:** Hung Chan, Shan Zhao, Lin Zhang, Jeffery Ho, Czarina C.H. Leung, Wai T. Wong, Yuanyuan Tian, Xiaodong Liu, Thomas N.Y. Kwong, Raphael C.Y. Chan, Sidney S.B. Yu, Maggie H.T. Wang, Gary Tse, Sunny H. Wong, Matthew T.V. Chan, William K.K. Wu

**Affiliations:** ^1^ Department of Anaesthesia and Intensive Care The Chinese University of Hong Kong Hong Kong Hong Kong; ^2^ Department of Medicine & Therapeutics The Chinese University of Hong Kong Hong Kong Hong Kong; ^3^ State Key Laboratory of Digestive Disease LKS Institute of Health Sciences The Chinese University of Hong Kong Hong Kong Hong Kong; ^4^ Department of Microbiology The Chinese University of Hong Kong Hong Kong Hong Kong; ^5^ School of Biomedical Sciences The Chinese University of Hong Kong Hong Kong Hong Kong; ^6^ The Jockey Club School of Public Health and Primary Care The Chinese University of Hong Kong Hong Kong Hong Kong

**Keywords:** autophagy, mechanistic target of rapamycin, mouse embryonic fibroblast, toxin B

## Abstract

Toxin B (TcdB) is a major pathogenic factor of *Clostridum difficile*. However, the mechanism by which TcdB exerts its cytotoxic action in host cells is still not completely known. Herein, we report for the first time that TcdB induced autophagic cell death in cultured human colonocytes. The induction of autophagy was demonstrated by the increased levels of LC3‐II, formation of LC3^+^ autophagosomes, accumulation of acidic vesicular organelles and reduced levels of the autophagic substrate p62/SQSTM1. TcdB‐induced autophagy was also accompanied by the repression of phosphoinositide 3‐kinase (PI3K)/Akt/mechanistic target of rapamycin (mTOR) complex 1 activity. Functionally, pharmacological inhibition of autophagy by wortmannin or chloroquine or knockdown of autophagy‐related genes Beclin 1, Atg5 and Atg7 attenuated TcdB‐induced cell death in colonocytes. Genetic ablation of *Atg5*, a gene required for autophagosome formation, also mitigated the cytotoxic effect of TcdB. In conclusion, our study demonstrated that autophagy serves as a pro‐death mechanism mediating the cytotoxic action of TcdB in colonocytes. This discovery suggested that blockade of autophagy might be a novel therapeutic strategy for *C. difficile* infection.

## 1 INTRODUCTION

*Clostridium difficile* is an anaerobic, Gram‐positive, sporulating bacterium that gives rise to a spectrum of gastrointestinal diseases, including antibiotic‐associated diarrhoea, pseudomembranous colitis and toxic megacolon.^1^ *C. difficile* infection is a major cause of hospital‐associated infection, accounting for about 3 million cases per year worldwide.^2^ It is also the most common infectious cause of diarrhoea in Intensive Care Unit in which the prevalence is estimated to be about 4%. One‐fifth of these ICU patients infected with *C. difficile* develop fulminant colitis with a mortality rate of nearly 60%.^3^ The incidence of *C. difficile* is rapidly increasing, especially in developed countries. Therefore, *C. difficile* infection, which carries significant mortality and morbidity, remains a major health burden. Several factors have been associated with an increased risk for *C. difficile* infection, including (i) recent exposure to antibiotics, such as clindamycin, fluoroquinolones and cephalosporins; (ii) the use of acid‐suppressive medications; (iii) recent hospitalization; (iv) advanced age and (v) comorbid conditions, such as the use of feeding tube and (vi) history of gastrointestinal surgery.^4^

The pathogenicity of *C. difficile* has been attributed to the 2 major exotoxins, namely Toxin A (TcdA) and Toxin B (TcdB), encoded by its genome. Both exotoxins can glycosylate and thereby inhibiting small GTPases of host cells to (i) mediate cytotoxicity; (ii) disrupt actin cytoskeletons and tight junctions and thus impair the epithelial barrier function; and (iii) promote the release of inflammatory mediators, such as interleukin‐8 and macrophage inflammatory protein 2. To this end, TcdB is 10 times more potent than TcdA in mediating the pathogenicity.^5^

Cessation of the inciting antibiotic and treatment with metronidazole and vancomycin are the mainstay in the management of *C. difficile* infection. Nevertheless, high rates of non‐responsiveness (~22%) and relapse (~27%) have been associated with metronidazole. In addition, the emergence of vancomycin‐resistant enterococci is a major concern for vancomycin.^3^ Several non‐antibiotic treatment modalities, such as toxin neutralization, probiotics and faecal microbiota transplantation, have been attempted, but their efficacies remain to be established. Development of other non‐antibiotic therapeutics for *C. difficile* infection is an area of active investigation.

Autophagy is a regulated intracellular degradation system, participating in various human diseases and physiological processes, such as cancer, neurodegeneration, microbial infection, ageing and heart disease.^6^, ^7^ Although autophagy could be a cellular protective mechanism especially in times of nutrient deprivation and other stressful conditions, its extensive activation could mediate cell death. The precise role of autophagy in *C. difficile*‐induced intestinal cell death and inflammation, however, remains unclear. In this study, we sought to investigate whether autophagy takes part in the cytotoxicity of TcdB in colonocytes.

## 2 MATERIAL AND METHODS

### 2.1 Colonocyte and mouse embryonic fibroblast culture

The human normal colonic epithelial cell line NCM460 was obtained from the American Type Culture Collection. *Atg5* ^*−/−*^ and wild‐type mouse embryonic fibroblasts (MEFs) were a gift from Dr. Noboru Mizushima at Tokyo Medical and Dental University, Japan. NCM460 and MEF cells were cultured in M3 medium and Dulbecco's modified Eagle's medium, respectively, supplemented with 10% foetal bovine serum and 1% penicillin‐streptomycin at 37°C in 5% CO_2_.

### 2.2 Cytotoxicity assay

Cells were incubated in the absence or presence of different concentrations of TcdB followed by CCK‐8 (2‐(2‐methoxy‐4‐nitrophenyl)‐3‐(4‐nitrophenyl)‐5‐(2,4‐disulfophenyl)‐2H‐tetrazolium, monosodium salt) cytotoxicity assay for cell viability detection. In brief, cells were seeded at a density of 6000 cells per well in 96‐well plates. CCK‐8 solution dissolved in the culture medium at the final volume of 10 μL/well was added to each well at 48 hours, and the plates were measured at 450 nm using a microplate reader.

### 2.3 Western blots

After incubation with TcdB of different concentrations at the, NCM460 cells were harvested, washed with ice‐cold 1× phosphate‐buffered saline (PBS) and lysed in immunoprecipitation assay buffer [150 mmol L^−1^ NaCl, 50 mmol L^−1^ Tris, 2 mmol L^−1^ ethyleneglycol‐bis(β‐aminoethylether), 2 mmol L^−1^ EDTA, 25 mmol L^−1^ NaF, 25 mmol L^−1^ β‐glycerophosphate, 0.2% Triton X‐100, 0.3% Nonidet P‐40 and 0.1 mmol L^−1^ phenylmethylsulfonyl fluoride]. Cellular debris was pelleted by centrifugation at 13 000 *g* for 30 minutes at 4°C. The concentrations of the total lysate protein were measured using a standard Bradford assay (Bio‐Rad, San Diego, CA, USA). For Western blots, 10 μg of protein from the total cell lysate was electrophoresed by SDS‐PAGE. The proteins were then transferred to nitrocellulose membrane (Pierce Chemical) and probed with primary antibodies followed by horseradish peroxidase‐labelled secondary antibodies. Proteins were visualized using enhanced chemiluminescence (Pierce Chemical).

### 2.4 RNA interference

Cells were seeded at a density of 8000 cells per well in 96‐well plates prior to the transfection. During the transfection, the cells were transfected with small interfering RNA (Thermo Fisher) against autophagy‐related genes (ie Beclin 1, Atg5 and Atg7) using the jetPRIME transfection reagent (Polyplus) according to the manufacturer's instructions.

### 2.5 Detection of LC3^+^ autophagosomes

NCM460 cells were grown on glass chamber slides overnight and then transfected with mCherry‐GFP‐LC3 for 24 hours. After transfection, cells were exposed to TcdB (10 ng/mL) for different periods of time. Cells were then rinsed twice with 1× PBS and fixed in 4% paraformaldehyde for 15 minutes at room temperature. After rinsing twice with 1 ×  PBS, the slides were mounted in prolong gold anti‐fade reagent (Invitrogen, Carlsbad, CA, USA) and then analysed on a confocal microscope (Leica).

### 2.6 Acridine orange staining for acidic vesicular organelles

NCM460 cells were exposed to TcdB (10 ng/mL) for different periods of time. After washing once with 1× PBS, acridine orange was added at a final concentration of 1 μg/mL for a period of 15 minutes. Pictures were obtained with a fluorescent microscope equipped with a 450‐490‐nm band‐pass blue excitation filters, a 505‐nm dichroic mirror, a 520‐nm long pass‐barrier filter.

### 2.7 Quantitative PCR

Total RNA was extracted by Trizol and then reverse‐transcribed into complementary DNA by a PrimeScript^™^ RT reagent Kit (TaKaRa). p62/SQSTM1 mRNA expression was measured by quantitative PCR with SYBR Pre‐mix Ex Taq kit (TaKaRa) using the following primers: p62/SQSTM1 forward: AGGCGCATACCGCGAT; reverse: CGTCACTGGAAAAGGCAACC. 28S gene expression was used as an endogenous control. The expression level of p62 mRNA was calculated using the delta delta *C*_T_ method.

### 2.8 Terminal deoxynucleotidyl transferase dUTP nick end labelling (TUNEL) staining

Cells were incubated in the absence or presence of different concentrations of TcdB with staurosporine (1 μmol L^−1^) as a positive control for different periods of time. Afterwards, cells were subsequently determined by TUNEL assay with In Situ Cell Death Detection Kit, TMR red (Roche), according to the manufacturer׳s protocol. Micrographs were captured by a confocal microscope (Leica).

### 2.9 Statistical analysis

Statistical analysis was performed with one‐way analysis of variance (ANOVA) followed by the Tukey's post hoc test. *P* values <.05 will be considered statistically significant.

## 3 RESULTS

### 3.1 TcdB reduces viability of colonocytes without inducing apoptosis

**Figure 1:**
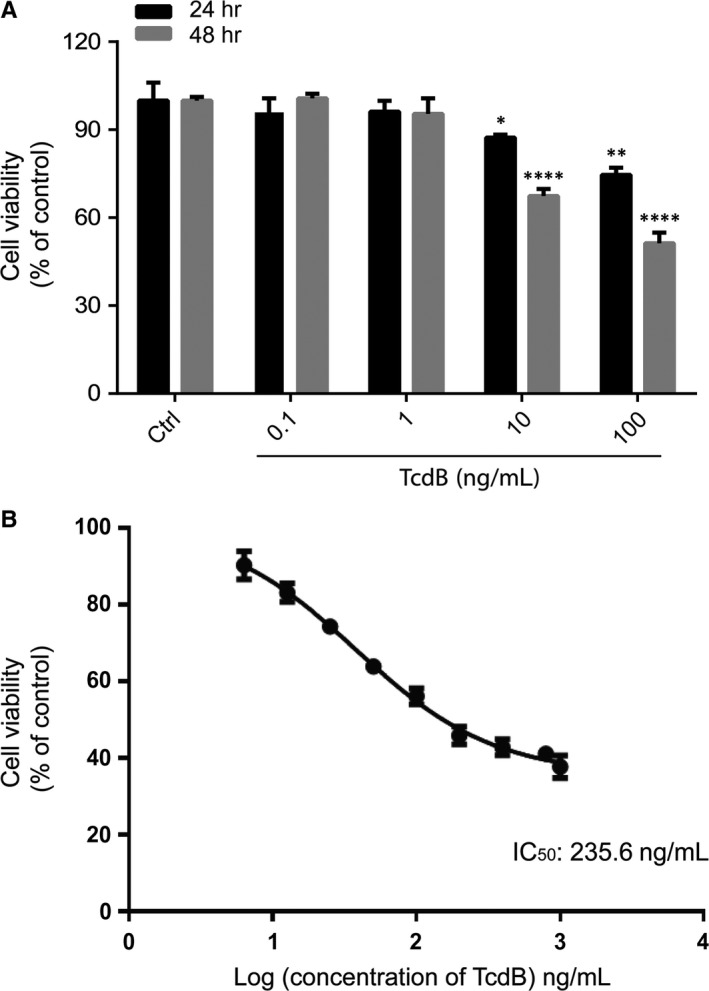
Cytotoxicity of TcdB in cultured human colonocytes. A, Time‐course and dose‐dependent effect of TcdB‐induced cell death in NCM460. NCM460 cells were cultured with the indicated concentrations (0.1‐100 ng/mL) of TcdB for 24 or 48 h. Cell viability was assessed by CCK‐8 assay. Results are expressed as percentage of control at corresponding time‐points and represent the means ± SEM of 3 independent experiments. *P <* *.05; **P <* *.01; ****P <* *.0001 significantly different between groups. B, Dose‐response curve assessing cell viability in NCM460 cells after TcdB exposure. NCM460 cells were cultured with the indicated concentrations (0.1‐1000 ng/mL) for 48 h. Cell viability was assessed by CCK‐8 assay

**Figure 2:**
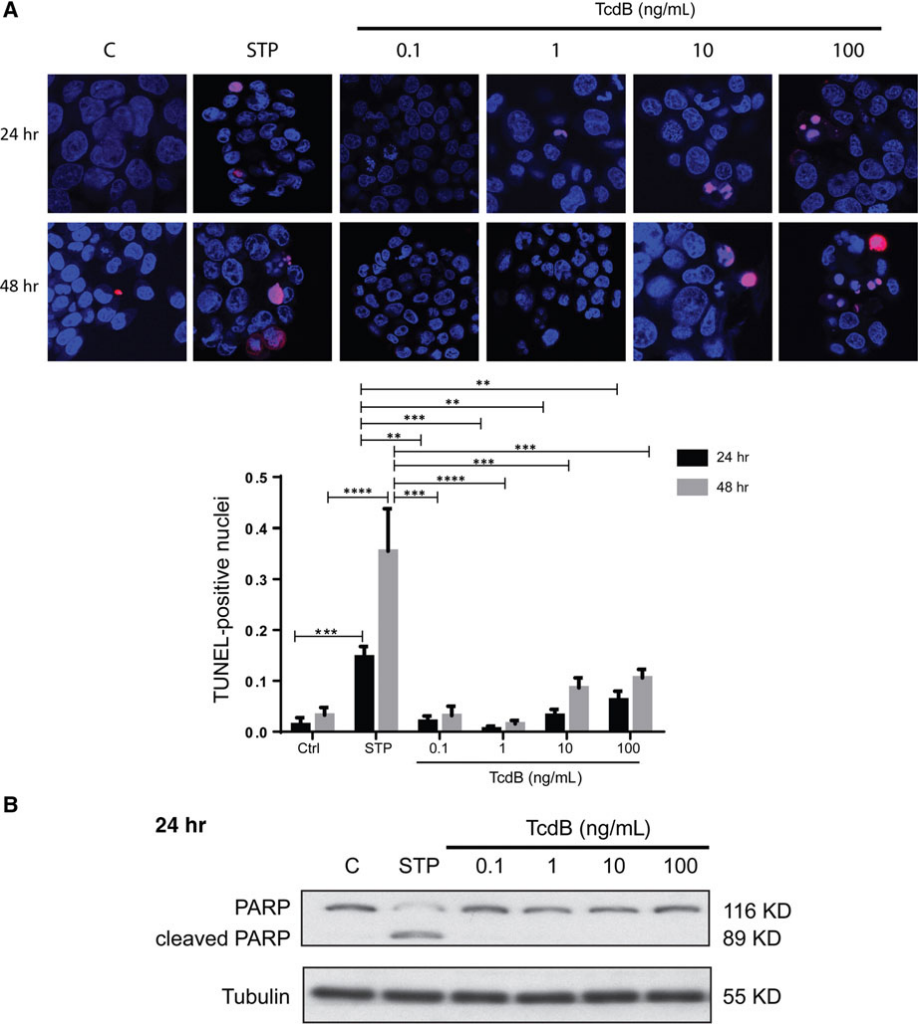
Apoptosis is not the primary cell death mechanism of TcdB‐exposed colonocytes. A, NCM460 cells were cultured with the indicated concentrations (0.1‐100 ng/mL) of TcdB for 24 or 48 h. DNA fragmentation of apoptotic cells was assessed by TUNEL assay. Quantitative data represent means ± SEM of 3 independent experiments. **P <* *.01; ***P <* *.001; ****P <* *.0001 significantly different between groups. B, Exposure to TcdB (10 ng/mL) did not induce PARP cleavage in NCM460 cells. Staurosporine (STP; 1 μmol L^−1^), a known apoptosis inducer, was used as the positive control. Representative blots of 3 independent experiments are shown

Colon is the primary anatomic site of *C. difficile* infection. We therefore determined the effect of TcdB on the viability of cultured human colonocytes. NCM460 cells were exposed to increasing concentrations of TcdB from 0.1 to 100 ng/mL for 24 or 48 hours followed by the CCK‐8 assay. As shown in Figure 1A, TcdB reduced the viability of NCM460 cells in a dose‐ and time‐dependent manner. The significant cytotoxic effect of TcdB could be detected at the concentration as low as 10 ng/mL at the 24‐hour time‐point. Dose‐response analysis for assessing the half maximal inhibitory concentration (IC_50_) of TcdB was also conducted. It was found that the IC_50_ of TcdB in NCM460 cells was 235.6 ng/mL at the 48‐hour time‐point. To assess whether TcdB reduced viability of NCM460 cells, TUNEL staining was performed. As shown in Figure 2A, TcdB exposure for 24 or 48 hours did not cause substantial DNA fragmentation as compared with the known apoptotic inducer staurosporine. Moreover, TcdB failed to induce PARP‐1 cleavage (Figure 2B), suggesting that apoptosis was not the primary cell death mechanism in TcdB‐exposed colonocytes.

### 3.2 TcdB induces autophagy in colonocytes

**Figure 3:**
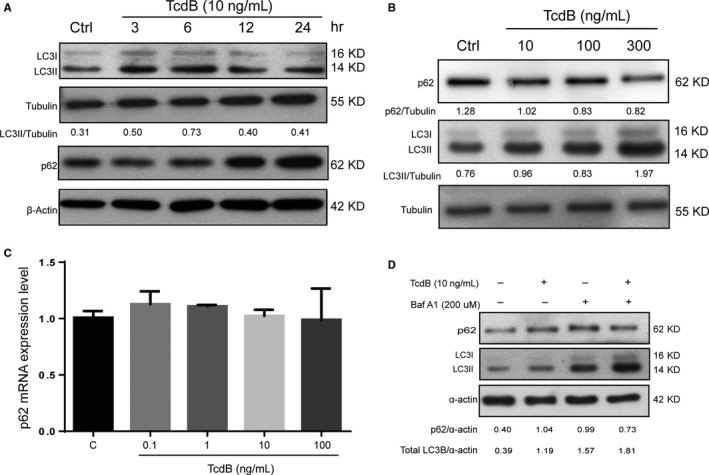
Increased autophagosome formation in colonocytes exposed to TcdB. A, Exposure to TcdB at 10 ng/mL increased LC3‐II levels in NCM460 cells with the effect peaked at 6 h. B, TcdB increased the level of LC3‐II and degradation of p62/SQSTM1 in a concentration‐dependent manner. NCM460 cells were cultured with the indicated concentrations (10‐300 ng/mL) of TcdB for 6 h. C, TcdB‐mediated p62/SQSTM1 protein down‐regulation was independent of p62/SQSTM1 gene transcription. NCM460 cells were cultured with the indicated concentrations (0.1‐100 ng/mL) of TcdB for 6 h. D, NCM460 cells were exposed to TcdB (10 ng/mL) in the absence or presence of bafilomycin A1 (Baf A1; 200 μmol L^−1^) for 6 h. Representative blots of 3 independent experiments are shown

To further investigate the cell death mechanism underlying the cytotoxicity of TcdB in NCM460 cells, autophagy was assayed. First, the level of LC3B‐II as a biomarker of autophagosome formation was determined in TcdB‐exposed cells. As shown in Figure 3A, the induction of LC3B‐II level by low‐concentration TcdB (10 ng/mL) peaked at 6 hours and lessened afterwards. These results indicated that TcdB at low concentration could readily induce autophagy in NCM460 cells. Similarly, at the 6‐hour time‐point, higher concentrations of TcdB up to 300 ng/mL increased the levels of LC3B‐II (Figure 3B), during which autophagic flux was found to be increased as indicated by the reduced level of p62/SQSTM1 (an autophagic substrate). Importantly, TcdB‐mediated p62/SQSTM1 down‐regulation was not dependent of p62 gene transcription (Figure 3C). However, it is also noteworthy that the autophagic flux might be impaired afterwards as indicated by the increased level of p62/STSTM1 at the 24‐hour time‐point. To further investigate how TcdB regulated autophagic flux at the early time‐point (ie 6 hours), the late‐stage autophagy inhibitor bafilomycin A1 (BA1) was used. As shown in Figure 3D, treatment with BA1 (which blocks lysosomal acidification and the fusion between autophagosome and lysosome) did not prevent the induction of LC3B‐II levels by TcdB, suggesting that TcdB induced LC3B‐II level through promoting autophagosome formation instead of affecting late‐stage autophagy.

**Figure 4:**
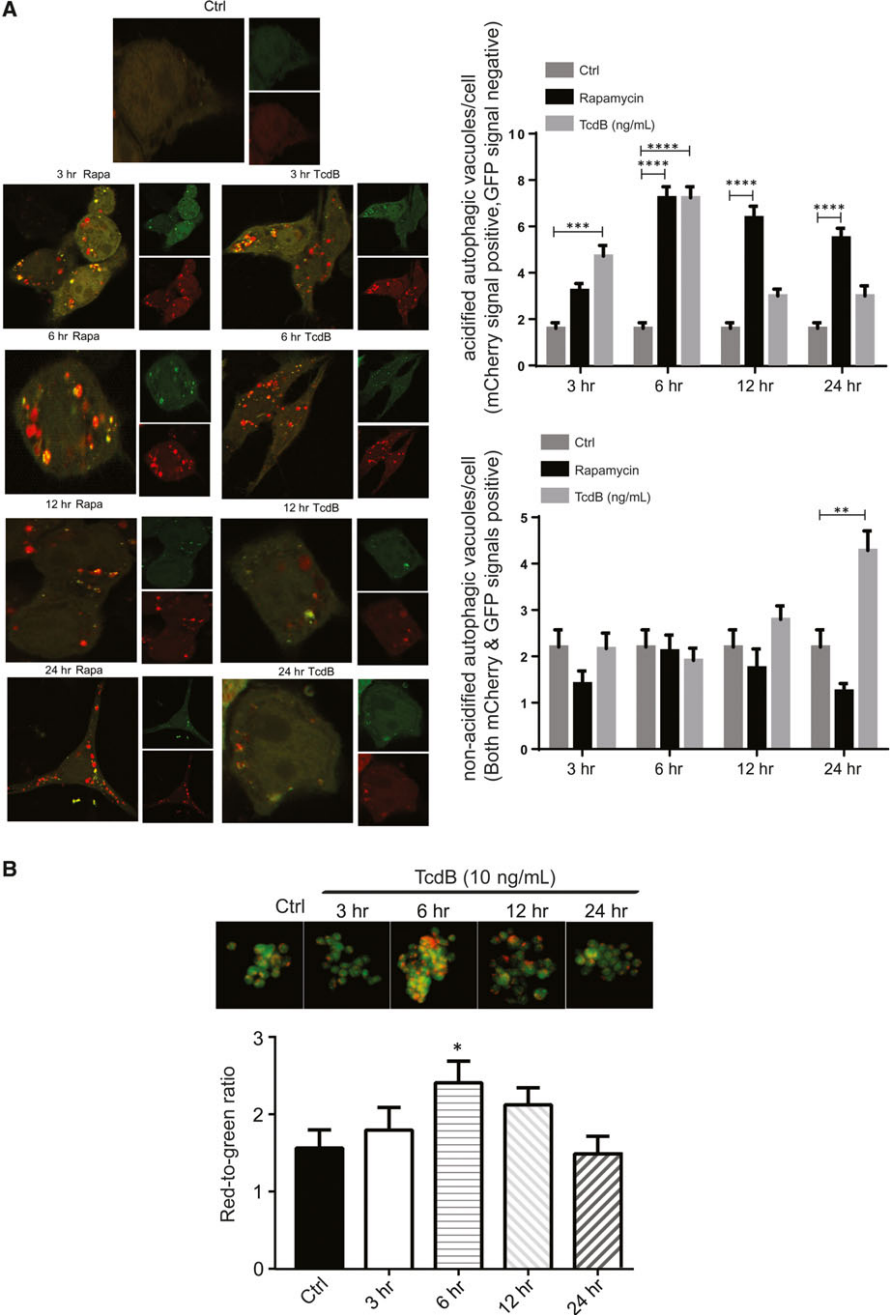
Enhanced formation of acidic vesicular organelles and autophagic flux in TcdB‐exposed colonocytes. A, NCM460 cells were transfected with mCherry‐GFP‐labelled LC3 plasmid for 24 h followed by exposure to TcdB (10 ng/mL) or rapamycin (1.1 μmol L^−1^). Acidified and non‐acidified LC3‐positive autophagosomes were visualized and counted under a confocal microscope. Quantitative data represent means ± SEM of 3 independent experiments. **P <* *.01; ***P <* *.001; ****P <* *.0001 significantly different between groups. B, Acridine orange staining was performed to visualize TcdB‐induced formation of acidic vesicular organelles (orange colour) in NCM460 under fluorescence microscopy. Quantitation of the red‐to‐green ratio represents means ± SEM of 3 independent experiments. *P <* *.05 significantly different between groups

To confirm the increased formation of autophagosomes upon TcdB exposure, NCM460 cells were transfected with the plasmid of mCherry‐GFP‐LC3B. A classical autophagy inducer rapamycin was used as a positive control. Both TcdB (10 ng/mL) and rapamycin significantly increased the number of mCherry‐positive, GFP‐negative LC3 puncta (acidified autophagic vacuoles) at the 6‐hour time‐point (Figure 4A). Concordant with the Western blot results for p62/SQSTM1, TcdB‐exposed cells exhibited impaired autophagic flux at the 24‐hour time‐point, as indicated by the accumulation of non‐acidified autophagic vacuoles (both mCherry‐positive and GFP‐positive).

Aside from the formation of LC3^+^ autophagosomes, increased staining of acidic vesicular organelles, including late endosomes, autolysosomes and lysosomes, is another important hallmark of autophagy. In this study, acridine orange, which emits bright red fluorescence in acidic intracellular compartments, was used to visualize putative acidic vesicular organelles. Colorimetric analysis demonstrated that TcdB (10 ng/mL) exposure resulted in a significant increase in red‐to‐green ratio in acridine orange‐stained cells (Figure 4B) at 6 hours, indicating the acumination of acidic vesicular organelles.

### 3.3 PTEN/PI3K/AKT/mTOR pathway is involved in TcdB‐induced autophagy

**Figure 5:**
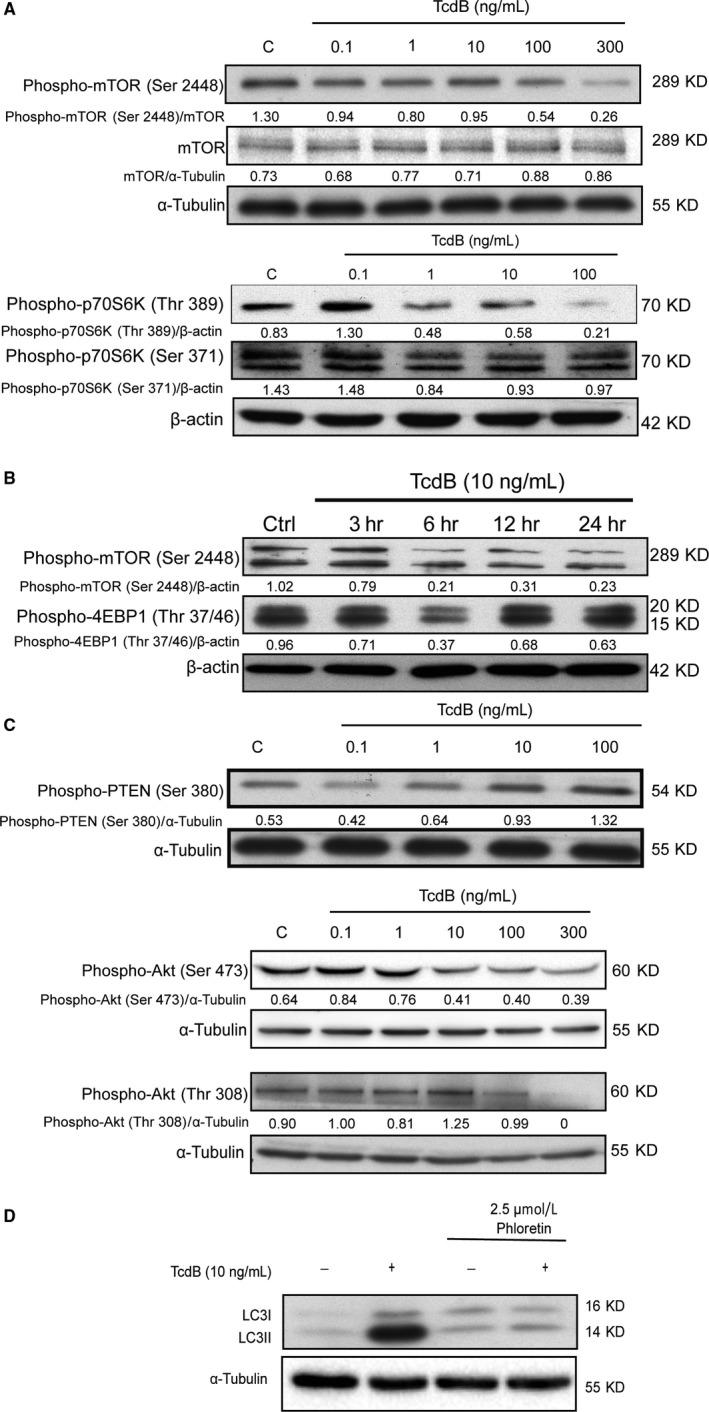
Inhibition of PTEN‐PI3K‐AKT‐mTOR complex 1 pathway by TcdB in colonocytes. A, NCM460 cells were exposed to TcdB for 6 h at the indicated concentrations. Phosphorylation of mTOR (Ser 2448) and p70‐S6K (Thr 389 and Ser 371) was determined by Western blots. B, Time‐course analysis of mTOR phosphorylation upon TcdB exposure was performed. C, TcdB suppressed PI3K/Akt pathway as shown by increased the phosphorylation of PTEN (Ser 380) and decreased phosphorylation of Akt (Ser 473 and Thr 308). D, Glucosyltransferase activity is required in TcdB‐triggered autophagy. NCM460 cells were exposed to TcdB in the absence or presence of the glucosyltransferase inhibitor phloretin (2.5 μmol L^−1^) for 6 h at the indicated concentrations. Representative blots of 3 independent experiments are shown

The mechanistic target of rapamycin (mTOR) complex 1 is a well‐known negative regulator of autophagosome formation. The activity of mTOR complex 1 could be assessed by the phosphorylation levels of mTOR itself and its downstream substrates, such as p70‐S6 kinase (p70‐S6K). To determine whether TcdB‐induced autophagy was associated with mTOR complex 1 repression, the phosphorylation levels of these 2 proteins were measured in NCM460 cells with or without exposure to TcdB. As shown in Figure 5A, TcdB dose‐dependently reduced the phosphorylation levels of mTOR at Serine 2448 and p70‐S6K at Threonine 389 and Serine 371. The time‐dependent reduction in mTOR phosphorylation at Serine 2448 by TcdB was also demonstrated (Figure 5B). To further elucidate the mechanism by TcdB‐mediated mTOR inhibition, we studied the potential involvement of the PTEN‐PI3K‐Akt pathway, which is an important signalling cascade upstream of mTOR in regulating cell survival, cell growth and initiation of autophagy. Phosphatase and tensin homologue (PTEN) is a negative regulator of PI3K‐AKT‐mTOR pathway. Activation of PTEN triggers inactivation of Akt and subsequently inactivates mTOR signalling.^8^ As shown in Figure 5C, TcdB concentration‐dependently increased the phosphorylation level of PTEN at Serine 380 and subsequently reduced the phosphorylation levels of Akt at Serine 473 and Threonine 308.

### 3.4 Toxin glucosyltransferase activity is required for the pro‐autophagic effect of TcdB

TcdB is known to exert its cytotoxic action through its glucosyltransferase activity,^9^ which subsequently leads to cell rounding and cell death. To examine the critical role of glucosyltransferase activity in TcdB‐induced autophagy, a non‐competitive glucosyltransferase inhibitor phloretin^10^ was used. As shown in Figure 5D, phloretin inhibited the TcdB‐mediated induction of LC3B‐II level.

### 3.5 Pharmacological or genetic ablation of autophagy attenuates TcdB‐induced cell death

**Figure 6:**
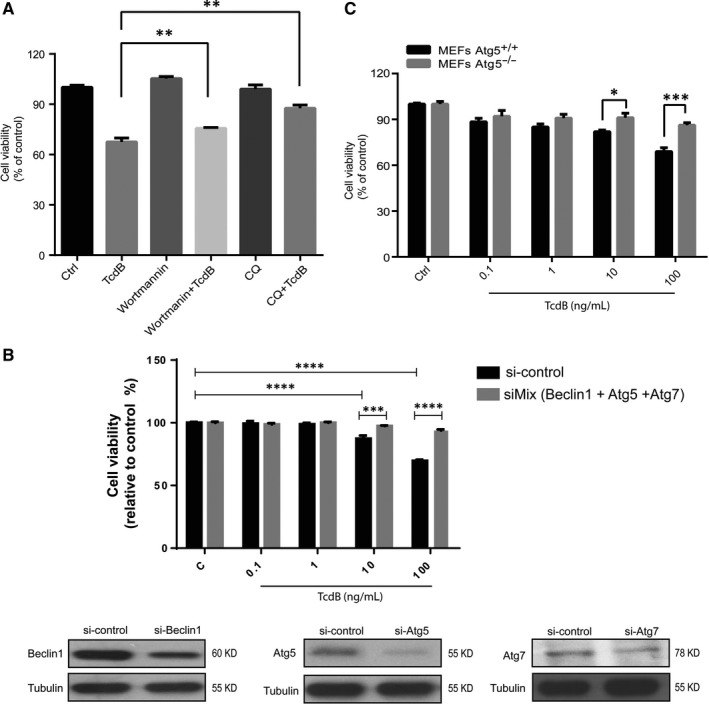
Attenuation of the cytotoxic effect of TcdB by autophagy inhibition. A, NCM460 cells were exposed to TcdB (10 ng/mL) in the absence or presence of wortmannin (Wort; 200 nmol L^−1^) or chloroquine (CQ; 20 μmol L^−1^) for 48 h. B, NCM460 cells were transfected with control siRNA or mixture of siRNAs targeting Beclin 1, Atg5 and Atg7 before the exposure to TcdB at the indicated concentrations for 48 h. Cell viability was assessed by CCK‐8 assay. C, Wild‐type and *Atg5* knockout mouse embryonic fibroblasts (MEFs) were exposed to TcdB at the indicated concentrations for 48 h. Cell viability was assessed by CCK‐8 assay. Results are expressed as means of percentage of control ± SEM of 3 independent experiments. *P <* *.05; **P <* *.01; ***P <* *.001; ****P <* *.0001 significantly different between groups

Autophagy could function as a pro‐survival or pro‐death mechanism depending on biological context. To examine the role of autophagy in TcdB‐induced cell death, 2 different approaches were used to inhibit autophagy. First, autophagy was inhibited pharmacologically using wortmannin (200 nmol/L, a Class I/III phosphoinositide 3‐kinase inhibitor) and chloroquine (20 μmol L^−1^, a lysosomotropic agent that inhibits lysosome‐autophagosome fusion and lysosomal protein degradation). Both agents partially reversed TcdB‐induced cell death in NCM460 cells (Figure 6A), suggesting that autophagy was required for the cytotoxicity of TcdB in colonocytes. Moreover, the pro‐death function of TcdB autophagy was verified with siRNA‐mediated knockdown of autophagy‐related genes (Atg5, Atg7 and Beclin 1) in NCM460 cells. Simultaneous knockdown of Beclin 1, Atg5 and Atg7 alleviated the cell death induced by TcdB (10 and 100 ng/mL at the 48‐hour time‐point) (Figure 6B). Similar results were also found in murine embryonic fibroblasts (MEFs) derived from wild‐type and *Atg5* knockout mice. Atg5 encodes an E3 ubiquitin ligase necessary for autophagy due to its role in autophagosome elongation. As compared with the wild‐type MEFs, the loss of cell viability induced by TcdB (10 and 100 ng/mL at the 48‐hour time‐point) was mitigated by *Atg5* knockout (Figure 6C), confirming the pro‐death nature of TcdB‐induced autophagy.

## 4 DISCUSSION

*Clostridium difficile* infection is a vital cause of hospital‐associated infection worldwide and in locality. Secretion of 2 exotoxins, TcdA and TcdB, were regarded as the primary associated virulence determinants. Up to now, there exist 2 paradoxes about the potential functions of exotoxins. One is that purified TcdA without TcdB could bring obvious symptoms of *C. difficile* infection, but TcdB alone could not have the effect except it is combined with TcdA.^11^ Another opinion, based on results from a hamster model, is that TcdB is vital for *C. difficile* virulence while TcdA was avirulent.^12^ As the problems of unclear infection mechanism and emerging antibiotic resistance are serious, *C. difficile* infection has become more difficult to treat. Investigating the pathogenic mechanism would therefore help to devise new therapeutic approaches.

Previous studies have shown that TcdB is a potent cytotoxin capable of inducing enzyme‐independent necrosis in both cells and tissue.^13^ In this study, we focus on the role of autophagy. As autophagy is a complicated and dynamics process, various methods are required for more accurate analysis in monitoring autophagy. Acridine orange is one of the methods to examine the formation of acidic compartments during autophagy. However, staining with acridine orange by itself is not a sufficient method for monitoring autophagy because it primarily detects lysosomes while an increase in lysosome size or number could reflect an increase in non‐professional phagocytosis instead of autophagy. For this reason, we have used other methods, such as mCherry‐GFP‐LC3 puncta formation assays and Western blots for autophagy markers, to demonstrate for the first time that TcdB could induce autophagic cell death in a dose‐ and time‐dependent manner in cultured human colonocytes. The loss of cell viability is associated with the induction of autophagy as demonstrated by increased LC3‐II levels, enhanced formation of LC3‐positive autophagic vacuoles, the accumulation of acidic vesicular organelles and reduced levels of the autophagic substrate p62/SQSTM1. Importantly, inhibition of autophagosome formation by pharmacological blockade, knockdown of Beclin 1, Atg5 and Atg7 or genetic ablation of *Atg5* attenuated the cytotoxicity of TcdB, suggesting that autophagy mediated at least in part the cytotoxicity. Although autophagy has been generally reported as an essential mechanism for the maintenance of cell survival, especially in times of nutrient deprivation and other stressful conditions, which could protect host cells against various forms of cellular insults, extensive activation of autophagy could mediate cell death.

mTOR complex 1 signalling pathway serves as a major player in transmitting the autophagic signals. In *Saccharomyces cerevisiae*, inhibition of TOR is required to control the Atg1‐dependent organization of pre‐autophagosomal elements and the expansion of the pre‐autophagosomal membrane.^14^, ^15^ In humans, inhibition of mTORC1 has been reported to induce autophagy in different biological context. For instances, mTOR inhibitors induce autophagy in lung cancer, glioma, mantle cell lymphoma and disseminated gastric cancer cells.^16^, ^17^, ^18^, ^19^ In this study, we demonstrated that TcdB suppressed mTORC1 activity as demonstrated by reduced phosphorylation of mTOR and its substrate p70‐S6K. We further demonstrated that TcdB suppressed mTOR complex 1 activity through the PTEN‐PI3K‐AKT‐mTOR pathway as indicated by increased phosphorylation of PTEN and the reduced phosphorylation of Akt at Serine 473 and Threonine 308. However, the precise upstream mechanism by which TcdB regulates the PTEN‐PI3K‐AKT‐mTOR pathway is still unclear as TcdB could mediate its signal through targeting multiple small GTPases, such as RhoA, Rac1 and Cdc42. Nevertheless, in this study, the glucosyltransferase activity of TcdB was found to be required for its pro‐autophagic effect.

The current findings implicate that the use of autophagy inhibitor might attenuate the virulence of TcdB. Further animal studies would be required to assess the in vivo therapeutic effect of autophagy inhibition during *C. difficile* infection.

## CONFLICT OF INTEREST

None to declare.
